# Immunohistochemical Patterns in the Interfollicular Caucasian Scalps: Influences of Age, Gender, and Alopecia

**DOI:** 10.1155/2013/769489

**Published:** 2013-12-22

**Authors:** Claudine Piérard-Franchimont, Geneviève Loussouarn, Ségolène Panhard, Didier Saint Léger, Myriam Mellul, Gérald E. Piérard

**Affiliations:** ^1^Department of Dermatopathology, University Hospital of Liège, 4000 Liège, Belgium; ^2^Laboratory of Skin Bioengineering and Imaging, Department of Clinical Sciences, University of Liège, 4000 Liège, Belgium; ^3^L'Oréal, Research & Innovation, 93400 Saint-Ouen, France; ^4^DSL Consult, 75000 Paris, France

## Abstract

Skin ageing and gender influences on the scalp have been seldom studied. We revisited the changes in the interfollicular scalp. The study was performed on a population of 650 volunteers (300 women and 350 men) for over 7 years. Three age groups were selected in both genders, namely, subjects aged 20–35, 50–60, and 60–70 years. The hair status was further considered according to nonalopecic and alopecic patterns and severity (discrete, moderate, and severe). Biopsies from the parietal area were processed for immunohistochemistry. Stromal cells were distinguished according to the presence of vimentin, Factor XIIIa, CD117, and versican. Blood and lymphatic vessels were highlighted by Ulex europaeus agglutinin-1 and human podoplanin immunoreactivities, respectively. Actinic elastosis was identified by the lysozyme coating of elastic fibres. The epidermis was explored using the CD44 variant 3 and Ki67 immunolabellings. Biplot analyses were performed. Immunohistochemistry revealed a prominent gender effect in young adults. Both Factor XIIIa+ dermal dendrocytes and the microvasculature size decreased with scalp ageing. Alopecia changes mimicked stress-induced premature senescence.

## 1. Introduction

Contrasting with other body regions, the scalp has received little attention regarding changes possibly occurring in its specific structure during life span in both genders. The histo-pathological aspect of the scalp is quite different from the skin of any other part of the body, but such regional variability is rarely taken into consideration in the overall assessment of skin ageing. This process globally results from the combination of a series of intrinsic and extrinsic factors. Among them, seven main influences were distinguished including genetic, chronological, actinic, behavioural, endocrine, catabolic, and gravitational effects [1]. Scalp photoaging, inside and outside bald areas, has been seldom studied [2–4]. The clinical expression of scalp ageing apparently shows less interindividual variations than on other parts of the body. Little information is available about chronological ageing of the scalp and hair, except when senescent alopecia develops [5, 6].

The present study focused on possible variations in the structures of the interfollicular part of the reticular dermis and epidermis according to gender, age, and alopecia status.

## 2. Volunteers and Methods

### 2.1. Volunteers

The study was approved by the University Hospital Ethics Committee. A total of 650 healthy non-smoker Caucasian phototypes III-IV subjects with indoor occupations (300 women and 350 men) were enrolled over a period of 7 years. None recalled previous and recurrent episodes of dandruff. Participants were interviewed by means of a questionnaire covering the past medical history and the current status of the scalp condition. Subjects were stratified into subgroups according to age, gender, and absence or presence of alopecia (Table 1). In volunteers with alopecia, no systemic disturbance was observed including altered amounts of circulating hormones and biological parameters recognized being involved in hair loss. During skin surgery procedure for a benign tumour of the scalp (melanocytic naevus, seborrhoeic keratosis, epithelial cyst, etc.), a 6 mm punch biopsy was further performed after local anesthesia on the parietal area that looked healthy at the clinical inspection.

### 2.2. Immunohistochemistry and Image Analysis

Microscopical sections (6 *μ*m thick) were cut from the formalin-fixed paraffin-embedded punch biopsies. The sections were used for immunohistochemical assessments using a panel of antibodies (Table 2) and the avidin-biotin peroxidase method. Following a 1 h incubation time with any of the primary antibodies, the slides were washed in Tris-buffered saline (TBS) and incubated for 30 min with the secondary antibody (biotinylated swine anti-rabbit, 1 : 300, Dakopatts). The slides were rinsed in TBS and covered by the EnVision (Dakopatts, Glostrup, Denmark) polymer-based revelation system. Following TBS washings, Fast Red (Dakopatts) was used as chromogen substrate. The last steps dealt with counterstaining with Mayer's haemalum before mounting. Negative immunohistochemical controls were performed by omitting or substituting the primary and the secondary antibodies of the laboratory procedure.

Computerized image analysis (Analysis Olympus, Tokyo, Japan) was applied on fields at ×250 magnification of the interfollicular dermis and epidermis, showing absence of inflammatory cell infiltration. Data about each antibody were pooled in each volunteer before calculation of the relevant value expressed per mm length of the section. The numbers of Ki67+, Factor XIIIa+, and vimentin+ cells were determined. In practice, the numbers of immunoreactive spots larger than 20 *μ*m^2^ were counted. This procedure allowed identification of the main cytoplasmic core of cells while excluding fragments of dendritic extensions. Relative areas were measured for the structures identified by CD44 variant 3, human podoplanin, lysozyme, Ulex europaeus agglutinin-1 (UEA-1), and versican. With regard to blood vessels, the area delimited by the annular lining of endothelial cells was measured. The sum of these vascular lumens was divided by the area of the reticular dermis under consideration for determining a relative area covered by the microvasculature.

### 2.3. Statistical Analysis

The Shapiro-Wilk test was used to test normality of the data distributions. Means, standard deviations, medians, and ranges were calculated, when appropriate, for each variable. The two-sided Student paired *t*-test was used for comparing data showing normal distributions. The Mann-Whitney *U* test was performed when Gaussian distributions were not achieved. In these assessments, *P* < 0.05 was considered as statistically significant. Percentages of variation were calculated between paired groups of data. Multiple combinations of data were presented as radars. Biplot analysis was used as a graphical tool to present results from principal component analysis. It corresponded to a scatterplot displaying a rank-2 matrix [7, 8].

## 3. Results

**Figure 1:**
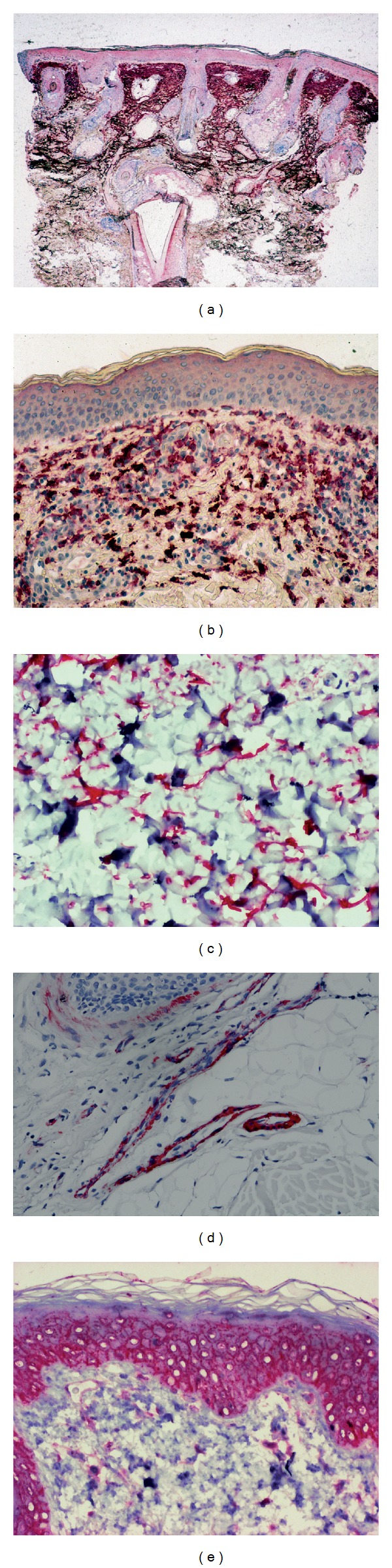
Typical aspects of immunohistological labeling. (a) Lyzozyme in severe solar elastosis; (b) Versican in dermal cells; (c) Versican in dendritic dermal cells; (d) Ulex europaeus agglutinin-1 in endothelial cells of blood vessels; (e) CD44 varismt 3 in keratinocytes and dermal cells.

All volunteers had fair skin and various hair colours. They recalled less than one scalp sunburn a year during the whole lifetime. Immunohistochemical findings are summarized in Table 3, distinguishing the effects of gender, age, and alopecia on the composition of the interfollicular reticular dermis and epidermis. Typical immunohistochemical aspects are depicted in Figure 1.

### 3.1. Gender Influence on the Scalp of Young Adults without Alopecia

Gender-related differences for each immunolabelling are given in Table 3. A significant larger amount in solar elastosis (lysozyme+) and a significant increase in dermal cellularity (vimentin+) and dermal dendrocytes (Factor XIIIa+) were found in men (*P* < 0.001). Other gender-related differences were observed in the microvasculature. The blood (UEA-1+) and lymphatic (podoplanin+) vasculatures as well as the numerical density in CD117+ mast cells were significantly more developed in women (*P* < 0.001).

Although the number of Ki67+ keratinocytes engaged in the cell cycle of proliferation was similar in both genders, the stratum Malpighi decorated by the antibody to CD44 variant 3 was significantly thicker in men (*P* < 0.001). The epidermal CD44 immunolabelling was uniform in all subjects.

**Figure 2:**
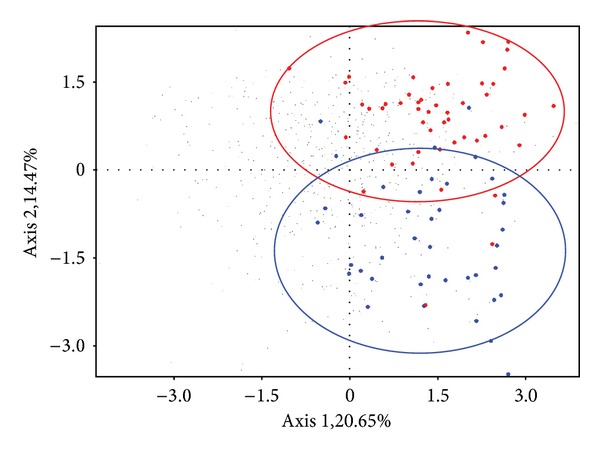
Biplot analysis comparing young individuals without alopecia. Men (*∙*) and women (▲) are clearly distinguished by principal component analysis.

The biplot analysis, considering the whole immunohistochemical information from all young subjects without alopecia, revealed a strong distinction between genders (Figure 2).

### 3.2. Age Effect on the Scalp of Subjects without Alopecia

**Figure 3:**
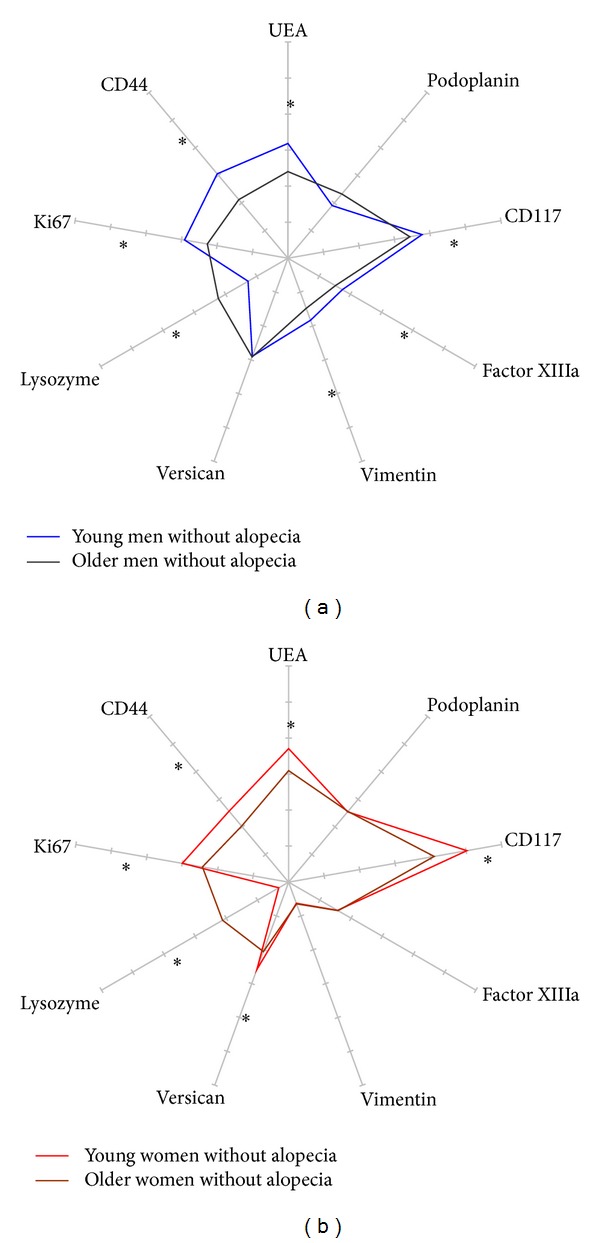
Radars of 9 immunostainings in the interfollicular scalp in young (■) and older (■) subjects with hair fullness. (a) Men; (b) women.

The influence of age on the scalp structure was assessed comparing, in each gender unaffected by alopecia, the young adults on the one hand and all subjects over 50 years of age on the other hand (Table 3, Figures 3(a) and 3(b)).

In men, the most prominent age-related changes consisted in a significant (*P* < 0.001) increased amount of lysozyme-coating elastotic fibres and a significant reduction (*P* < 0.001) in the size of vascular lumens lined by UEA-1+ endothelial cells. With ageing, the density in vimentin+ cells significantly decreased, while dermal dendrocytes (Factor XIIIa+) increased in a similar magnitude. Both Ki67+ and CD44+ variant 3 immunolabellings in the epidermis significantly (*P* < 0.001) decreased with age.

In women, lysozyme deposits in the dermis were found markedly increased (*P* < 0.001) with age. The immunolabellings for versican, UEA-1+ vasculature, and CD117+ mast cells were similarly reduced (*P* < 0.001) with age. The epidermis showed similar reductions in Ki67 and CD44+ variant 3 immunolabelling (*P* < 0.001).

**Figure 4:**
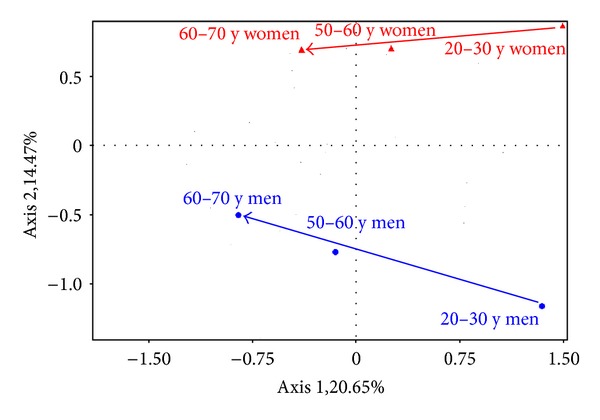
Biplot analysis showing the age effect on the interfollicular dermis in both genders.

The ways of global changes experienced during scalp ageing are illustrated on a biplot analysis (Figure 4), showing that age-induced modifications are quite similar for both genders, at the exception of a larger decrease of the cellularity in men.

### 3.3. Effect of Alopecia on the Scalp of Young Adults

**Figure 5:**
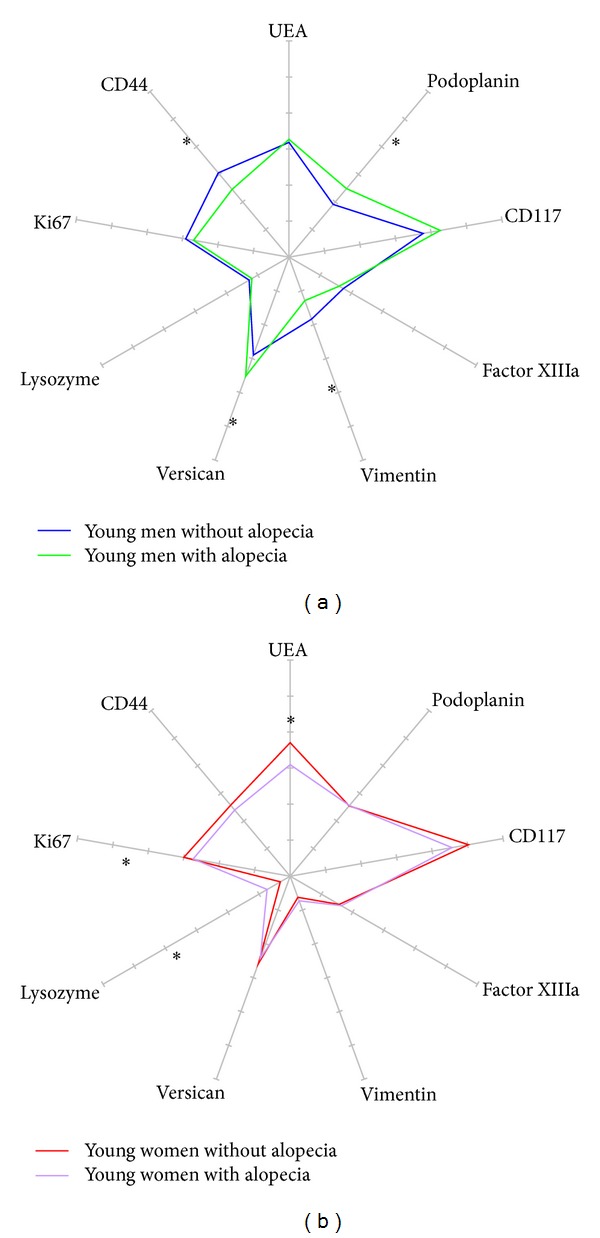
Radars of 9 immunostainings in the interfollicular scalp of young adults with (■) and without (■) alopecia. (a) Men; (b) women.

The impact of alopecia on the interfollicular scalp was assessed in young adults of both genders (Table 3, Figures 5(a) and 5(b)). In men, alopecia was associated with a significant (*P* < 0.001) increase in the amounts of versican+ stromal cells and human podoplanin+ lymphatic vessels. The epidermis was significantly (*P* < 0.001) thinner, as observed with the CD44 variant 3 immunolabelling. In women, a significant (*P* < 0.001) increase in lysozyme deposits on elastotic fibres was the most prominent effect, correlating with alopecia.

### 3.4. Combined Influences of Age and Alopecia on the Scalp

**Figure 6:**
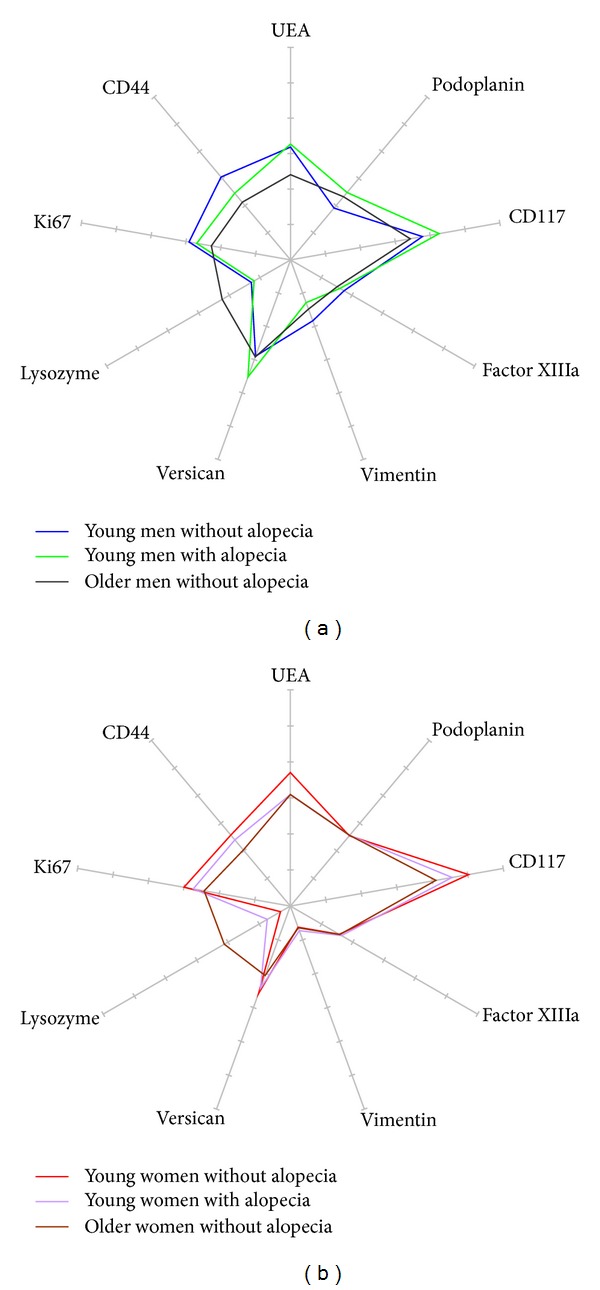
Radars of 9 immunostainings in the interfollicular scalp of subjects of different ages and alopecia status: (■) young without alopecia, (■) young with alopecia, (■) older without alopecia. (a) Men; (b) women.

The combined influences of age and alopecia in both genders are shown in radars forms (Figures 6(a) and 6(b)). In a biplot analysis, the directions of global changes occurring with alopecia in women appeared to be moderately different from the ageing process (Figure 6), whereas the two phenomena were quite different in men.

## 4. Discussion

Skin ageing is a multifaceted process influenced by various factors, affecting different body sites at variable extents [1]. The interfollicular scalp has not been thoroughly explored with regard to possible changes with age, gender, and alopecia. By contrast, the pilosebaceous follicle has attracted more attention in its involvement in hair growth during ageing and alopecia [6, 9, 10], leading to a better understanding of the human hair follicle from birth to senescence. The goal of the present study was to investigate and analyse findings associated with interfollicular scalp ageing in relation with gender and possible androgenic or senescent alopecia. No change has been reported yet in the literature about the interfollicular reticular dermis. We did not consider microinflammatory foci that commonly accompany alopecia [11–15]. Similarly, we discarded the typical changes found at the site of former hair follicles in androgenic alopecia [2, 11, 13]. Indeed, in this scalp condition, inflamed streamers and fibrous tracts extend downwards from the hair follicle [16, 17]. The present data refer to the parietal scalp area and cannot be extrapolated with confidence to the whole scalp.

### 4.1. Gender Effect

The present study suggests a gender difference in the number of vimentin+ dermal cells. To the best of our knowledge, the higher cell density disclosed in the scalp of young men has never been reported so far. It is acknowledged that a number of phenotypical and functional differences do exist between distinct vimentin+ dermal cells. Among them, versican+ cells are quite abundant and appear to be uniformly distributed throughout the dermis. Versican is a large proteoglycan normally present inside stromal cells of the skin. This molecule belongs to the chondroitin sulphate family of the hyalectan group, named for its ability to bind hyaluronan [18]. The volume occupied by versican+ cells did not show any gender difference in subjects without alopecia. In these individuals, we did not observe an increase in versican with age as previously reported on sun-shielded skin of the buttocks [19].

Another group of vimentin+ cells is represented by dermal dendrocytes recognized by their immunoreactivity for Factor XIIIa [20]. Similar to vimentin+ stromal cells, they appeared more abundant in men. They are preferentially found adjacent to the superficial microvasculature [20, 21]. Circumstantial evidence indicates that some interactions exist between Factor XIIIa+ dermal dendrocytes and mast cells [22]. In the present study, the relative abundance of Factor XIIIa+ dendrocytes in men was unexpectedly associated with a lower number in CD117+ mast cells. In addition, Factor XIIIa+ dendrocytes are possibly altered in their number and tensegrity by intrinsic mechanical forces present in the dermis [23–27]. The relationship between dermal dendrocytes and mast cells and the extent in the microvasculature network have only been previously scrutinized on body sites other than scalp [28].

Assessing skin microvasculature, using histopathology and immuno-histochemistry, proves to be difficult unless objective measurements are performed. Counting the number of transversal sections of vessels is unreliable due to vessel tortuosity overestimating the actual number of vessels. By contrast, measuring the size of the vascular lumens seems more realistic in the estimation of the vascular development. Euclidean and fractal analyses possibly bring different information [29]. The present findings confirm a reduction, over the years in the size of the vascular lumens lined by UEA-1+ cells [21]. Most conventional immunohistochemical methods for detecting endothelial cells do not distinguish lymphatics from blood vessels. In the present study, the UEA-1 immunolabelling predominantly highlighted blood vessels, and podoplanin identified lymphatic vessels [30, 31]. The vascular channels lined by UEA-1+ and podoplanin+ cells appeared larger in the scalp of women. This feature might be related to the functional activity of the increased numbers of CD117+ mast cells found in the scalp of women.

The extracellular matrix revealed abundant deposits of lysozyme in men. Lysozyme coating of elastic fibres is one of the best recognized immunohistopathological markers for dermal damages of chronic sun exposure [2, 32, 33]. This suggests that cumulative actinic damages were more pronounced. Hair length, styling, and density are likely involved in this feature.

The epidermis appeared thicker in men, although the Ki67+ germinative compartment appeared gender independent. By contrast, our findings suggest that the transit time of keratinocytes in the stratum Malpighi is possibly influenced by the gender, appearing slower in men.

### 4.2. Age Effect

The skin microvasculature is believed to shrink with age, although only limited objective quantitative data are available [20, 34]. Our findings suggest that the reduction in skin vascularity with ageing affects predominantly blood vessels rather than lymphatics.

A reduction in both the volume of versican+ cells and the number of CD117+ mast cells was particularly obvious in ageing women. These findings are consistent with an age-related reduction in dermal proteoglycans.

Previous studies have established the effect of ultraviolet (UV) light on the scalp, particularly when alopecia was present [2, 3, 35]. As expected, lysozyme deposits were more abundant with photoaging.

Consistent with previous reports, the thickness and cell production in the epidermis declined with ageing.

### 4.3. Effect of Alopecia

**Figure 7:**
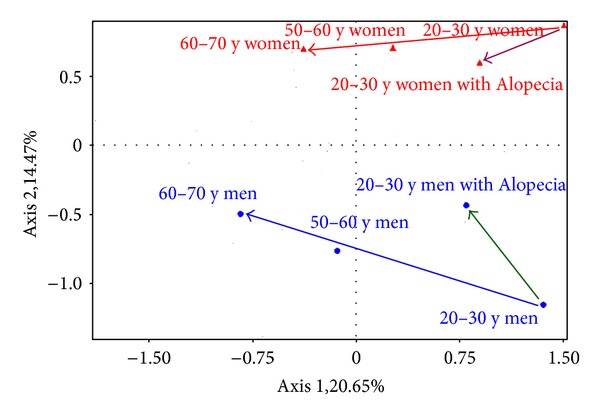
Biplot analysis showing distinct evolutions in the interfollicular dermis during the ageing and alopecia processes in men and women.

The effect of alopecia, if any, was only assessed in young adults for avoiding the confounding influence of ageing. As compared to people exhibiting hair fullness, a striking gender difference was found in alopecic subjects (Figure 7). Young alopecic men had fewer vimentin+ cells but significantly larger versican+ cells and enlarged podoplanin+ lymphatic vessels. By contrast, young alopecic women showed a prominent increase in lysozyme deposits on elastic fibres whereas the UEA-1 vasculature was reduced. In addition, their epidermal Ki67+ germinative pool was decreased. By contrast, the epidermis in alopecic men was thinner without any change in the Ki67 immunoreactivity. These findings clearly point out to two distinct gender-related hair disorders, as far as an interaction with the reticular dermis is concerned.

## 5. Conclusion

One strong aspect of the present study lies in the large array of immunohistochemical indicators of skin ageing used in a broad population. A chief finding is the unexpected gender difference in the interfollicular scalp condition of young adults without alopecia. On the whole, alopecia appears as one factor involved in the so-called stress-induced premature senescence of the interfollicular scalp [2, 35].

Basically, a tentative comparison between the present findings with descriptions on ageing glabrous skin suggests that typical signs of chronological ageing were blurred by those of dermal photoaging. Indeed, solar elastosis appeared as a hallmark of scalp ageing.

The present findings suggest a cross-talk between the structure of the interfollicular dermis and the activity of the hair cycle. The cause is unsettled but could correspond to the release of a variety of cytokines and growth factors with a possible intervention of mechanobiology. Indeed, the scalp is tightly bound to the underlying structures and hairs as well as to the dermal streamers at the site of former follicles in alopecia which are key elements controlling scalp tethering to deeper structures.

## Figures and Tables

**Table:** (a)

Women (number)			
Age (years)	Without alopecia	Moderate alopecia	Total
20–35	50	50	100
50–60	51	50	101
60–70	49	50	99

Total	150	150	300

**Table:** (b)

Men (number)				
Age (years)	Without alopecia	Moderate alopecia	Severe alopecia	Total
20–30	40	35	51	126
50–60	35	35	42	112
60–70	41	36	35	112

Total	116	106	128	350

**Table 2:** Panel of antibodies.

Antigen/antibody	Dilution	Source
CD44 variant 3	1 : 10	Menarini
CD117	1 : 100	Dako
Factor XIIIa	1 : 100	Neomarkers
Human podoplanin	1 : 100	Angiobio
Ki67	1 : 100	Dako
Lysozyme	1 : 300	Dako
Ulex europaeus agglutinin-1	1 : 2000	Sigma
Versican	1 : 500	Seikagaku corp.
Vimentin	1 : 200	Dako

**Table 3:** Percentage of variation (%) in the immunolabelling of the interfollicular scalp in males (M) and females (F), older (O) or younger (Y).

Tissue/antigen	Gender effect	Age effect		Alopecia impact	
	M versus F (%)	OM versus YM (%)	OF versus YF (%)	Alopecia versus hair fullness	
				YM (%)	YF (%)
Reticular dermis					
Lysozyme	305*	74*	571*	0	134*
Vimentin	193*	−19*	0	−30	0
Factor XIIIa	11*	19*	0	−7	5
Versican	0	0	−20*	22*	0
Ulex europaeus agglutinin	−14*	−25*	−17*	0	−16*
Podoplanin	−25*	0	0	30*	0
CD117	−25*	9*	−17*	0	0
Epidermis					
CD44 variant 3	19*	−30*	−21*	−22*	0
Ki67	0	−22*	−19*	0	−9*

*P < 0.001.

## References

[1] Piérard GE (2004). Ageing across the life span: time to think again. *Journal of Cosmetic Dermatology*.

[2] Piérard-Franchimont C, Uhoda I, Saint-Léger D, Piérard GE (2002). Androgenic alopecia and stress-induced premature senescence by cumulative ultraviolet light exposure. *Exogenous Dermatology*.

[3] Trüeb RM (2003). Is androgenetic alopecia a photoaggravated dermatosis?. *Dermatology*.

[4] Xhauflaire-Uhoda E, Piérard-Franchimont C, Piérard GE, Quatresooz P (2010). Weathering of the hairless scalp: a study using skin capacitance imaging and ultraviolet light-enhanced visualization. *Clinical and Experimental Dermatology*.

[5] Kligman AM (1988). The comparative histopathology of male-pattern baldness and senescent baldness. *Clinics in Dermatology*.

[6] Trüeb RM (2005). Aging of hair. *Journal of Cosmetic Dermatology*.

[7] Gabriel KR (1971). The biplot graphic display of matrices with application to principal component analysis. *Biometrika*.

[8] Gabriel KR (2002). Goodness of fit of biplots and correspondence analysis. *Biometrika*.

[9] Malgouries S, Donovan M, Thibaut S, Bernard BA (2008). Heparanase 1: a key participant of inner root sheath differentiation program and hair follicle homeostasis. *Experimental Dermatology*.

[10] Malgouries S, Thibaut S, Bernard BA (2008). Proteoglycan expression patterns in human hair follicle. *British Journal of Dermatology*.

[11] Jaworsky C, Kligman AM, Murphy GF (1992). Characterization of inflammatory infiltrates in male pattern alopecia: implications for pathogenesis. *British Journal of Dermatology*.

[12] Piérard-Franchimont C, De Doncker P, Camvenbergh G, Pierard GE (1998). Ketoconazole shampoo: effect of long-term use in androgenic alopecia. *Dermatology*.

[13] Mahé YF, Michelet J, Billoni N (2000). Androgenetic alopecia and microinflammation. *International Journal of Dermatology*.

[14] Piérard-Franchimont C, Xhauflaire-Uhoda E, Loussouarn G, Saint Léger D, Piérard GE (2006). Dandruff-associated smouldering alopecia: a chronobiological assessment over 5 years. *Clinical and Experimental Dermatology*.

[15] Piérard-Franchimont C, Xhauflaire-Uhoda E, Piérard GE (2006). Revisiting dandruff. *International Journal of Cosmetic Science*.

[16] Sellheyer K, Bergfeld WF (2006). Histopathologic evaluation of alopecias. *American Journal of Dermatopathology*.

[17] El-Domyati M, Attia S, Saleh F, Abdel-Wahab H (2009). Androgenetic alopecia in males: a histopathological and ultrastructural study. *Journal of Cosmetic Dermatology*.

[18] Wight TN (2002). Versican: a versatile extracellular matrix proteoglycan in cell biology. *Current Opinion in Cell Biology*.

[19] Quatresooz P, Paquet P, Hermanns-Lê T, Piérard GE (2008). Molecular mapping of factor XIIIa-enriched dendrocytes in the skin (review). *International Journal of Molecular Medicine*.

[20] Quatresooz P, Piérard GE (2009). Immunohistochemical clues at aging of the skin microvascular unit. *Journal of Cutaneous Pathology*.

[21] Sueki H, Whitaker D, Buchsbaum M, Murphy GF (1993). Novel interactions between dermal dendrocytes and mast cells in human skin: implications for hemostasis and matrix repair. *Laboratory Investigation*.

[22] Penneys NS, Rademaker B, Jackson IT, Fasching MC (1991). Loss of factor XIIIa in pig dermis during tissue expansion. *Journal of Dermatological Science*.

[23] Hermanns-Lê T, Uhoda I, Piérard-Franchimont C, Piérard GE (2002). Factor XIII a-positive dermal dendrocytes and shear wave propagation in human skin. *European Journal of Clinical Investigation*.

[24] Silver FH, Siperko LM, Seehra GP (2003). Mechanobiology of force transduction in dermal tissue. *Skin Research and Technology*.

[25] Quatresooz P, Hermanns-Lê T, Ciccarelli A, Beckers A, Piérard GE (2005). Tensegrity and type I dermal dendrocytes in acromegaly. *European Journal of Clinical Investigation*.

[26] Piérard GE, Piérard-Franchimont C, Hermanns-Lê T, Quatresooz P (2007). Polishing up functional dermatopathology, dermometrology and mechanobiology. *Revue Medicale de Liege*.

[27] Quatresooz P, Piérard-Franchimont C, Piérard GE (2009). Vulnerability of reactive skin to electric current perception. A pilot study implicating mast cells and the lymphatic microvasculature. *Journal of Cosmetic Dermatology*.

[28] Uhoda I, Piérard GE, Piérard-Franchimont C (2005). Vascularity and fractal dimension of the dermo-epidermal interface in guttate and plaque-type psoriasis. *Dermatology*.

[29] Henry F, Quatresooz P, Valverde-Lopez JC, Piérard ǴE (2006). Blood vessel changes during pregnancy: a review. *American Journal of Clinical Dermatology*.

[30] Ordóñez NG (2006). Podoplanin: a novel diagnostic immunohistochemical marker. *Advances in Anatomic Pathology*.

[31] Albrecht S, From L, Kahn HJ (1991). Lysozyme in abnormal dermal elastic fibers of cutaneous aging, solar elastosis and pseudoxanthoma elasticum. *Journal of Cutaneous Pathology*.

[32] Park PW, Biedermann K, Mecham L, Bissett DL, Mecham RP (1996). Lysozyme binds to elastin and protects elastin from elastase-mediated degradation. *Journal of Investigative Dermatology*.

[33] Kelly RI, Pearse R, Bull RH, Leveque J-L, De Rigal J, Mortimer PS (1995). The effects of aging on the cutaneous microvasculature. *Journal of the American Academy of Dermatology*.

[34] Quatresooz P, Xhauflaire-Uhoda E, Piérard-Franchimont C, Piérard GE (2009). Epidermal field carcinogenesis in bald-headed: an attempt at finetuning early non-invasive detection. *Oncology Reports*.

[35] Toussaint O, Medrano EE, von Zglinicki T (2000). Cellular and molecular mechanisms of stress-induced premature senescence (SIPS) of human diploid fibroblasts and melanocytes. *Experimental Gerontology*.

